# Use of angiotensin-converting enzyme inhibitors immediately after stroke: Commentary

**DOI:** 10.4103/0972-2327.70872

**Published:** 2010

**Authors:** Subhash Kaul

**Affiliations:** Department of Neurology, Nizam Institute of Medical Sciences, Hyderabad, India

This issue of Annals of Indian Academy of Neurology features a debate on the controversial topic, ‘ACE inhibitors will help in improving stroke outcome if given immediately after stroke’. Dr. M.V. Padma favors the proposition, whereas, Dr. Rohit Bhatia opposes it. The topic is controversial because, while on the one hand the presence of severe hypertension in acute stroke is feared to be associated with the risk of developing cerebral edema, on the other hand antihypertensive treatment in acute stroke may lead to worsening of deficits due to reduced cerebral perfusion.[1] There are some studies, however, which suggest that no clinically significant change in cerebral perfusion occurs after the administration of Angiotensin Converting Enzyme (ACE) Inhibitors to patients soon after an ischemic stroke.[2,3] Dr. M.V. Padma emphasizes the fact that the mechanism of improvement in the stroke outcome may not be due to the effects of blood pressure reduction, but due to the endothelial-protective effects of the ACE Inhibitors, as shown in studies such as the Heart Outcomes Prevention Evaluation (HOPE) and Perindopril pRotection aGainst REcurrent Stroke Study (PROGRESS).[4,5] Dr. Rohit Bhatia convincingly rebuts this argument by commenting that the endothelial protective effects of ACE Inhibitors have been demonstrated only by their long-term use for stroke prevention and have not been tested in the setting of an acute stroke.[4,5] According to him there is a need for more studies to assess the theoretical effects of ACE Inhibitors on blood pressure, inflammatory cascade, and neuroprotection, in the acute phase of a stroke. He therefore strikes a cautionary note against going overboard in treating acute strokes with ACE Inhibitors, as there is not enough Class I evidence to support such practice at present. Dr. Padma, in support of her contention, cites studies that show that the use of ACE Inhibitors in the acute stage may improve the outcome. However, this inference was indirect, as the patients in these studies were already taking ACE Inhibitors at the onset of acute stroke and were not put on these drugs after the onset of the stroke.[6,7]

To strike a balance, as per the current evidence-based literature, there are concerns about instituting anti-hypertensive therapy immediately after a stroke, even if it is with ACE Inhibitors. Hypertension immediately after the stroke may be reactive and its presence may be necessary to compensate for the global and local autoregulatory failure following an acute stroke.[8] Even a mild reduction in blood pressure may be risky. The solution probably lies in taking a middle path. It seems prudent to withhold all antihypertensive medication for the first 24 hours after stroke onset, with some exceptions.[9] One may then cautiously and gradually begin to reduce the blood pressure, particularly in patients with underlying cerebrovascular atherostenotic lesions. The first choice should probably be ACE Inhibitors, pending the results of clinical trials testing other antihypertensive agents, in acute stroke.
